# The enzyme inhibitory activity of helminth-derived serine protease inhibitor rapidly establishes immune tolerance to ameliorate allergic diseases

**DOI:** 10.1080/21505594.2026.2725991

**Published:** 2026-09-06

**Authors:** Wenjie Shi, Nuo Xu, Yan Liu, Xue Bai, Xiaolei Liu, Ning Xu, Yi Liu

**Affiliations:** a State Key Laboratory for Diagnosis and Treatment of Severe Zoonotic Infectious Diseases, Key Laboratory for Zoonosis Research of the Ministry of Education, Institute of Zoonosis, and College of Veterinary Medicine, Jilin University, Changchun, China; b College of Public Health, Jilin Medical University, Jilin, China

**Keywords:** Trichinella spiralis, serpin, enzyme inhibitory activity, allergy, immune tolerance

## Abstract

Allergic diseases are a class of important immune imbalance diseases that lack effective cures. Helminth-derived serine protease inhibitors (serpins) exert immunoregulatory effects similar to those of helminth, are controllable, have few side effects, and have great application potential in correcting unbalanced immune responses. However, the immune regulatory effects and mechanisms mediated by the enzyme inhibitory activity of helminth-derived serpins have not been investigated or clarified in previous studies. We obtained mutant serpin proteins with significantly reduced enzyme inhibitory activity by predicting and mutating the single key amino acid. The therapeutic effect of immunoregulation mediated by enzyme inhibitory activity of serpin on allergic inflammation was investigated based on an acute allergy model constructed in OVA-specific T-cell receptor transgenic C57BL/6 mice. Unlike the Trichinella spiralis-derived serpin (Ts-serpin), the mutant proteins showed significantly reduced inhibitory activity against the chymotrypsin and elastase. Meanwhile, the immunosuppressive ability of the mutant proteins was weakened and mutant protein intervention groups showed more severe allergic inflammation in the lungs. Additionally, enzyme inhibitory activity regulates the differentiation of macrophages and Treg cells to establish immune tolerance, which is the key to rapidly improving lung injury at the challenge and treatment stages of allergy. These findings suggest that the rapid regulatory properties of enzymatic reactions have great potential in the prevention and treatment of allergic inflammation.

## Graphical abstract

**Figure:**
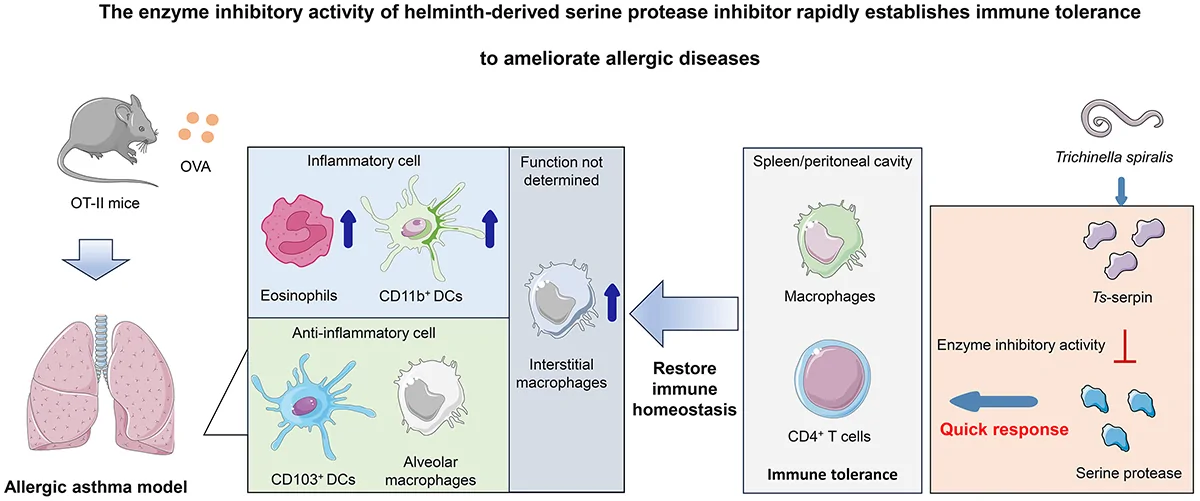

## Introduction

Allergy is an overreaction of the host immune system to harmless substances and is characterized by rapid onset and including condition such as allergic asthma, allergic rhinitis, allergic dermatitis, and food allergy [1,2]. At present, limited treatments for allergic diseases lead to recurrent attacks [3]. Correcting the imbalanced immune response and establishing an immune tolerance response may be an important way to cure allergic diseases [4]. Ovalbumin (OVA), a common allergen in humans and animals, is usually used to establish animal allergy models and is an important platform for studying the pathogenesis of allergies and screening therapeutic immunomodulators [5]. Since the concept of helminth-therapy was proposed, a variety of worms and their derived proteins have been demonstrated to ameliorate animal allergic diseases, including *Nippostrongylus brasiliensis* [6,7] and *Trichinella spiralis* (*T. spiralis*) [8,9]. The diverse immunoregulatory pathways of worms provide inspiration for the study of restoring an unbalanced immune system. Therefore, the excretory-secretory products (ESPs) on which worms exert immunomodulatory effects represent an important natural pharmacopoeia for screening immunomodulators [10,11]. Compared with live worm infection, natural proteins derived from worms have less toxic and fewer side effects [12,13], which makes them promising for clinical application.

*T. spiralis* is a parasitic nematode with a unique life history, and the immunomodulatory ability of adults plays an important role in inducing immune tolerance and inhibiting allergic inflammation [8]. In early infection, *T. spiralis* adults interfere with the host enzymatic reaction system [14,15], which is a rapid immunoregulatory mechanism. *T. spiralis*-derived serine protease inhibitors (*Ts*-serpin) is an immunomodulator that not only helps adults form an immune-tolerant microenvironment in the intestine, but also ameliorates colitis damage induced by TNBS [16]. Studies related to protease inhibitors usually confuse the immunoregulation mediated by protein structure with that mediated by enzyme inhibitory activity; thus, it is necessary to distinguish between these two pathways. To this end, we previously successfully constructed two mutant proteins with reduced enzyme inhibitory effects on chymotrypsin and elastase. Indeed, we found that *Ts*-serpin regulated the proliferation and differentiation of various immune cell subsets by inhibiting distinct serine proteases, which limited the active immune protective efficacy of the protein [17].

Here, this study aimed to explore the therapeutic efficacy and immunoregulatory mechanisms of the enzyme inhibitory activity of *Ts*-serpin in the OVA-induced allergy. We used *Ts*-serpin and mutant proteins to interfere with the three stages of the OVA-induced acute allergy model and evaluated the related efficacy and clarified the immunoregulatory cell network. Analysis of protein regulatory activity provides theoretical support for safe and accurate application of this protein in the treatment of allergic diseases in different states.

## Materials and methods

### Animals

6 ~ 8-week-old OVA-specific T-cell receptor transgenic C57BL/6 (OT-II) mice were purchased from the Model Animal Research Center, Nanjing University. The mice were bred in a specific pathogen-free facility and were provided with adequate water and food. The 60 mice were randomly divided into 12 groups of five mice and housed in individual cages (saline control, alum adjuvant control, OVA positive control, and protein intervention groups). Mice were anesthetized using isoflurane, and serum was collected through the tail vein. The mice were then euthanized by isoflurane combined with carbon dioxide inhalation. All animal experiments were performed in accordance with the guidelines for the care and use of laboratory animals [18]. The animal experiments were approved by the Ethical Committee of Jilin University which is affiliated with the Provincial Animal Health Committee (Changchun, China; Ethical clearance no.: DY202603030).

### Recombinant protein expression and purification

*Ts*-serpin-d1 and *Ts*-serpin-d2 were obtained by mutating *Ts*-serpin (GenBank: DQ864973.2) Thr_322_ and Ala_324_ to Glu, respectively. The amino acid mutation sites were located in the reactive center loop of the serpin and did not affect the protein structure. Recombinant proteins (r*Ts*-serpin/r*Ts*-serpin-d1/ r*Ts*-serpin-d2) were expressed by *Escherichia coli* BL21 (DE3) and purified using nickel column affinity chromatography. The purified recombinant proteins were analyzed using sodium dodecyl sulfate-polyacrylamide gel electrophoresis and Coomassie brilliant blue staining, and no obvious protein impurities were found.

### Enzyme inhibitory activity assays of the recombinant proteins

The process of detecting the enzyme inhibitory activity of proteins was consistent with previous studies [17]. In total, 20 μg of recombinant protein (r*Ts*-serpin/r*Ts*-serpin-d1/ r*Ts*-serpin-d2) was incubated with an equal amount of chymotrypsin or elastase for 10 min, followed by the addition of specific chromogenic substrates corresponding to the protease. The chymotrypsin chromogenic substrate was added to the mixture and incubated for 120 min, after which the absorbance at 410 nm was measured. The elastase chromogenic substrate was added to the mixture, which subsequently incubated for 15 min. Then, the absorbance was measured at 405 nm.

### Establishment of an acute allergy model and treatment with recombinant proteins

OT-II mice were sensitized intraperitoneally with 20 μg/50 μL OVA and Imject alum adjuvant in a total volume of 100 μL on Days 0, 7 and 14. As a control, the mice received Imject alum adjuvant or saline. The mice were challenged with aerosolized 1% OVA for 30 min on Days 28, 29 and 30 [8]. The protein treatments were delivered at 3 time points in the OVA-induced acute allergy model, including the sensitization stage, the challenge stage and the treatment stage. The sensitization-treated group received the 50 μg recombinant protein intraperitoneally on Days 0, 7, and 14. The challenge-treated group received the 50 μg recombinant protein intraperitoneally on Days 28, 29 and 30. The treatment- treated group received the 50 μg recombinant protein intraperitoneally on Days 31, 32 and 33. The inflammatory evaluation of the acute allergy model was performed on Day 37.

### Analysis of cells in bronchoalveolar lavage fluid

After the euthanized mice were bound, the chest cavity was dissected to expose the lung, and the right lungs were ligated with a surgical line. The neck skin was then dissected to expose the trachea. Then, 1 mL PBS was used to flush the lung repeatedly for 2 times to collect BALF [19]. BALF cells were fixed on slides and stained with DiffQuik according to the manufacturer’s instructions. In total, 200 cells were randomly counted, and the proportions of various immune cells, including macrophages, lymphocytes, eosinophils, and neutrophils, were determined according to their morphology.

### Lung histology

The ligated lung tissues of the mice were fixed in formalin and paraffin-embedded. The sections were stained with hematoxylin and eosin (H&E) or periodic acid-Schiff (PAS) and then scanned under an optical microscope.

### Detection of OVA-specific antibodies and cytokines in the serum

The serum samples were collected on Day 37 to detect OVA-specific IgG, IgG1 and IgE. The levels of OVA-specific IgG and IgG1 were detected with indirect ELISA. In brief, the OVA concentration was diluted to 5 μg/mL with antigen-coated buffer. Then, 100 μL of OVA buffer was added to the 96-well plates and incubated overnight at 4°C. After the plates were blocked with 5% skim milk for 1 h at 37°C, 1:500 diluted serum was added, and the mixture was incubated for 1 h at 37°C. Then, HRP-conjugated anti-mouse IgG and IgG1 antibodies were added and incubated for 1 h at 37°C. The results were obtained by the TMB reaction and a reading at 450 nm. OVA-specific IgE was detected with the mouse OVA-specific IgE ELISA kit according to the manufacturer’s instructions.

The TGF-β1 levels in the serum were detected with a commercial kit, and other cytokines were detected with Luminex.

### *In vitro* cell coculture experiment

The OT-II mice were euthanized and soaked in 75% alcohol for 10 min. The leg bones and spleens of the mice were collected in a sterile environment. The bone marrow cells were then treated with a 70 μm cell filter and red blood cell lysate. A total of 2 x 10^5^ cells were cultured with 1640 medium containing 10% fetal bovine serum in a 24-well plate supplemented with 25 ng/mL rmGM-CSF, and 10 ng/mL rmIL-4. After 2 and 4 days of culture, the culture medium was changed and the cytokines were supplemented. Suspended bone marrow-derived dendritic cells (BMDCs) were collected on Day 6 and incubated with 25 μg of OVA_323–339_ or 25 μg of r*Ts*-serpin/r*Ts*-serpin-d1/ r*Ts*-serpin-d2 for 24 h. After protein stimulation, the BMDCs were collected for subsequent cell coculture experiments.

The single-cell suspensions of the spleen were collected via mechanical grinding and a 70 μm cell filter. Mouse naïve CD4^+^ T cells were purified from single-cell suspensions with EasySep^TM^ mouse naïve CD4^+^ T-cell isolation kits according to the manufacturers’ instructions. A total of 2 x 10^5^ protein-treated BMDCs and 1 x 10^6^ naïve CD4^+^ T cells were cocultured for 48 h. The morphology of the cultured cells was observed under an optical microscope, and T-cell differentiation was detected via flow cytometry.

### Culture and adoptive transfer of bone marrow-derived macrophages (BMDMs)

BMDMs were isolated and cultured as described in the previous study [16]. In brief, mouse leg bones were isolated and bone marrow cells were collected via a 70 μm cell filter and red blood cell lysate. A total of 2 x 10^5^ cells were cultured with 1640 medium containing 10% fetal bovine serum in a 6-well plate supplemented with 25 ng/mL rmM-CSF. After 48 hours, the medium was changed and supplemented with rmM-CSF. After 96 hours, 50 μg of r*Ts*-serpin/r*Ts*-serpin-d1/ r*Ts*-serpin-d2 were added and the cells were incubated for 24 hours. In OVA-induced acute allergy model, 2 x 10^5^ protein-treated macrophages were injected intraperitoneally into mice during the challenge stage.

### Flow cytometry

The peritoneal cavity cells, spleen cells and lung cells of euthanized mice were collected on Day 37 for the analysis of immune cell subsets as described in a previous study [8,16]. First, 2 mL of precooled PBS was injected into the peritoneal cavity, and the abdominal cavity was repeatedly kneaded for 5 min. Then, PBS was collected and centrifuged to obtain peritoneal cavity cells. The cells were passed through a 70 μm filter, and 1 x 10^6^ cells were resuspended in 100 μL of PBS. The Fc block antibody was added, and the mixture was incubated for 10 min. Antibodies against F4/80, CD16/32, and CD206 were added, and the mixture was incubated for 30 min at 4°C. The stained cells were washed with PBS for 3 times and resuspended in 300 μL of PBS.

After the spleen cells were collected, red blood cell lysate was added, and the mixture was incubated for 10 min to remove erythrocyte. A total of 1 x 10^6^ cells were resuspended in 100 μL of PBS and stained with antibodies against cell surface marker molecules, including CD4, CD45, and B220, for 30 min at 4°C. After the cells were washed with PBS 3 times, the cells were resuspended in 100 μL of 4% paraformaldehyde and incubated for 30 min at 4°C. The cells were then suspended in 0.1% Triton X-100 and incubated for 40 min at 4°C to punch holes in the cell membrane. Next, the anti-mouse Foxp3 antibody was incubated with the cells for 30 min at 4°C. The stained cells were washed with PBS 3 times and resuspended in 300 μL of PBS.

The collected lung tissues were cut and treated with digestive fluid (HBSS containing 0.5 mg/mL collagenase IV and 8 μg/mL DNase I) for 40 min at 37°C. Lung single-cell suspensions were collected via a 70 μm cell filter and 1 x 10^6^ cells were resuspended in 100 μL of PBS for staining. The cells were stained with antibodies against cell surface marker molecules for 30 min at 4°C. CD11c, CD11b, Ly6G, and Siglec-F were used to identify eosinophils. CD11c, CD11b, CD24, MHC-II, and CD64 were used to identify alveolar macrophages, interstitial macrophages, CD11b^+^ DCs, and CD103^+^ DCs, respectively.

*In vitro* co-culture cells were collected and resuspended in 100 μL of PBS. Cells were stained with antibodies against cell surface marker molecules, including CD4, CD11c, CD86, MHCII. Cells were then incubated with anti-mouse Foxp3 antibody after 4% paraformaldehyde and 0.1% Triton X-100 treatment as described above. The stained cells were washed with PBS 3 times and resuspended in 300 μL of PBS.

### Statistical analysis

GraphPad Prism 5 software was used to analyze the differences in the experimental data and the results are shown as the means ± SDs. All the data are first analyzed using the normality and lognormality tests. The number of various immune cells in the BALF was statistically analyzed using two-way analysis of variance (ANOVA), and the OVA-positive group was compared with the other groups. The results are shown as follows: * *p* < 0.05; ** *p* < 0.01; *** *p* < 0.001. The statistical plots in the figure were generated via one-way analysis of variance (ANOVA) to compare the differences between the OVA-positive group and the other groups. The differences are shown as follows: * *p* < 0.05; ** *p* < 0.01; *** *p* < 0.001; ns, no significant difference. The statistical plots in the figure were generated via unpaired t tests to compare the differences between the *Ts*-serpin intervention group and the mutant protein intervention groups. The differences are shown as follows: # *p* < 0.05; ## *p* < 0.01; ### *p* < 0.001.

## Results

### *Ts*-serpin ameliorates allergic inflammation in lung tissue

**Figure 1.**
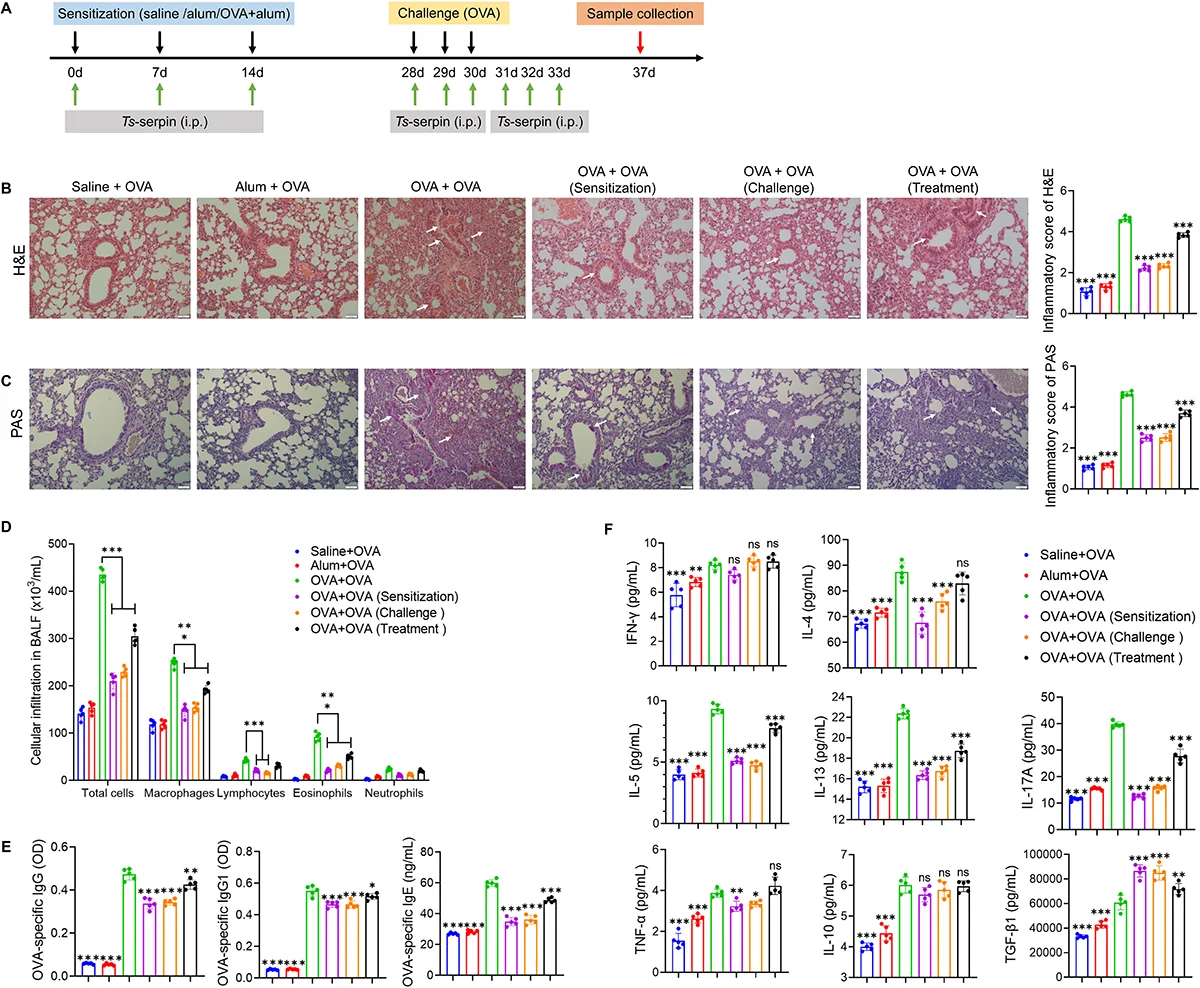
The treatment effects of *Ts*-serpin at three developmental stages of the OVA-induced acute allergy model.

To examine the effects of *Ts*-serpin intervention on the development of allergic diseases, we analyzed the effects of recombinant protein at the sensitization, challenge and treatment stages in the OVA-induced acute allergy model (Figure 1(A) and S1). OVA sensitization and challenge are prerequisites for inducing allergic inflammation in the mouse lung, which is characterized by inflammatory cell infiltration, thickening of bronchial walls, inflammatory exudates in bronchi, and goblet cell proliferation (Figure 1(B,C)). Compared with that in the OVA positive group, the inflammatory damage to the lung tissue in the *Ts*-serpin intervention groups was alleviated to different degrees, and the sensitization stage and challenge stage intervention groups presented the most obvious improvement (Figure 1(B,C)). Consistent with the improvement in lung tissue damage, the number of inflammatory cells, including macrophages, lymphocytes, and eosinophils, in the bronchoalveolar lavage fluid (BALF) was significantly reduced in the *Ts*-serpin intervention groups (Figure 1(D)).

(A) The intervention time points of *Ts-serpin* in different groups. (B) H&E staining results of mouse lung in the *Ts-serpin* intervention groups. Magnification: 200 x . (C) PAS staining results of mouse lung in the *Ts-serpin* intervention groups. Magnification: 200 x . (D) Statistical plot of the number of various immune cell subsets in the BALF. (E) The OVA-specific antibody levels (IgG, IgG1, IgE) in the serum. (F) The cytokine levels in the serum. The sample size for all data was five.

To assess the immune status of the mice, we examined the levels of OVA-specific antibodies and cytokines in the serum. The OVA-specific IgG/IgG1/IgE levels are low in OVA-unsensitized mice. The process of OVA sensitization was indispensable for the generation of specific antibodies (Figure 1(E)). After *Ts*-serpin intervention, the levels of OVA-specific antibodies decreased, and the intervention effects were most obvious at the sensitization stage and the challenge stage (Figure 1(E)). The results revealed that the levels of pro-inflammatory cytokines and anti-inflammatory cytokines, including IL-4, IL-5, IL-13, IFN-γ, IL-17A, TNF-α, IL-10, and TGF-β1, significantly increased in the OVA positive group compared with alum intervention group, and the increase in type 2 and Th17 immune-related cytokines was the most obvious (Figure 1(F)). Notably, the levels of type 2 and Th17 immune-related cytokines in the *Ts*-serpin intervention groups were significantly reduced. In addition, TGF-β1 levels were significantly increased, whereas the IL-10 and IFN-γ levels did not change (Figure 1(F)).

**Figure 2.**
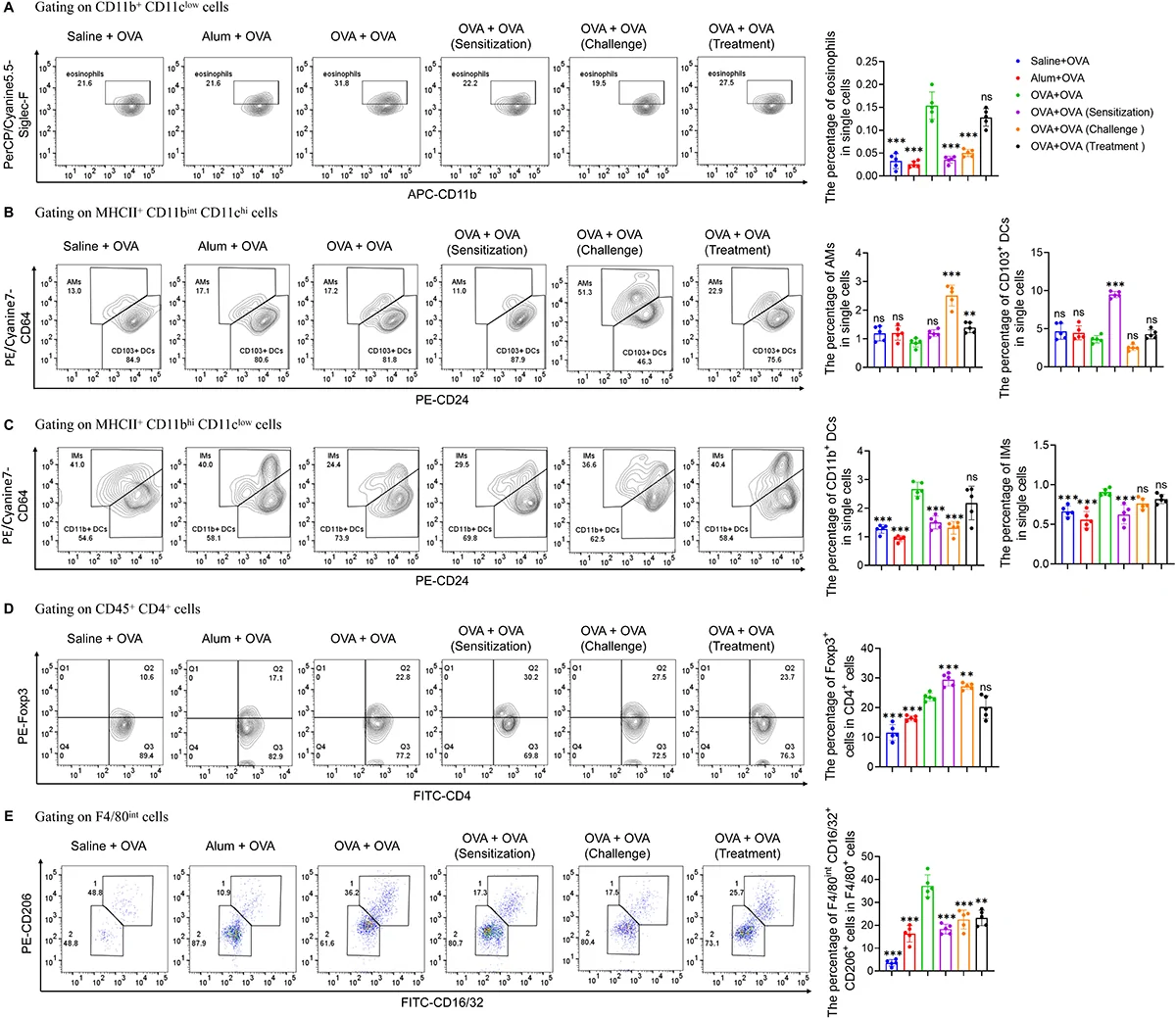
The regulatory effect of *Ts*-serpin on immune cells in different tissues at three developmental stages of OVA-induced acute allergy model.

To investigate potential cellular pathways involved in the cross-tissue immunoregulatory effects of these proteins in mice, we examined immune cell subsets in the mouse lung, spleen, and peritoneal cavity. The results revealed that the proportion of eosinophils in the lung tissue of the mice treated with the *Ts*-serpin in the sensitization and challenge stages was significantly lower than that in the OVA positive group, but no significant difference was noted in the treatment stage group (Figure 2(A)). *Ts*-serpin intervention in the sensitization stage increased the proportion of CD103^+^ DCs and decreased the proportions of CD11b^+^ DCs and interstitial macrophages (IMs). In contrast, *Ts*-serpin intervention at the challenge stage increased the proportion of alveolar macrophages (AMs) and reduced the proportion of CD11b^+^ DCs, whereas the *Ts*-serpin intervention in the treatment stage only slightly increased the proportion of AMs (Figure 2(B,C)). Additionally, *Ts*-serpin intervention did not affect the proportions of total CD45^+^ immune cells or B220^+^ CD4^−^ B cells in the spleen, but *Ts*-serpin intervention in the sensitization and challenge stages increased the proportion of regulatory T cells (Treg cells) (Figure 2(D) and S2(A-B)). Furthermore, *Ts*-serpin intervention did not affect the proportions of total F4/80^+^ macrophages, F4/80^hi^ macrophages, and F4/80^int^ macrophages in the peritoneal cavity, but induced F4/80^int^ macrophages presented low CD16/32 and CD206 expression levels (Figure 2(E) and S2(C-E)). Together, these data indicate that *Ts*-serpin inhibits lung tissue inflammation by enhancing the immune tolerance of mice.

(A) Plots and cell proportion statistics of eosinophils (CD11c^−^ CD11b^+^ SiglecF^+^) in the mouse lung. (B, C) Plots and cell proportion statistics of AMs (MHCII^+^ CD11c^hi^ CD11b^int^ CD24^−^ CD64^+^), CD103^+^ DCs (MHCII^+^ CD11c^hi^ CD11b^+^ CD24^+^ CD64^−^), IMs (MHCII^+^ CD11bhi CD24^−^ CD64^+^), and CD11b^+^ DCs (MHCII^+^ CD11b^hi^ CD24^+^ CD64^−^) in the mouse lung. (D) Plots and cell proportion statistics of CD4^+^ Foxp3^+^ Treg cells in the mouse spleen. (E) Plots and cell proportion statistics of F4/80^int^ CD16/32^+^ CD206^+^ macrophages in the mouse peritoneal cavity. The sample size for all data was five.

### The enzyme inhibitory activity of *Ts*-serpin slightly alleviates inflammation during intervention at the sensitization stage of the acute allergy model

**Figure 3.**
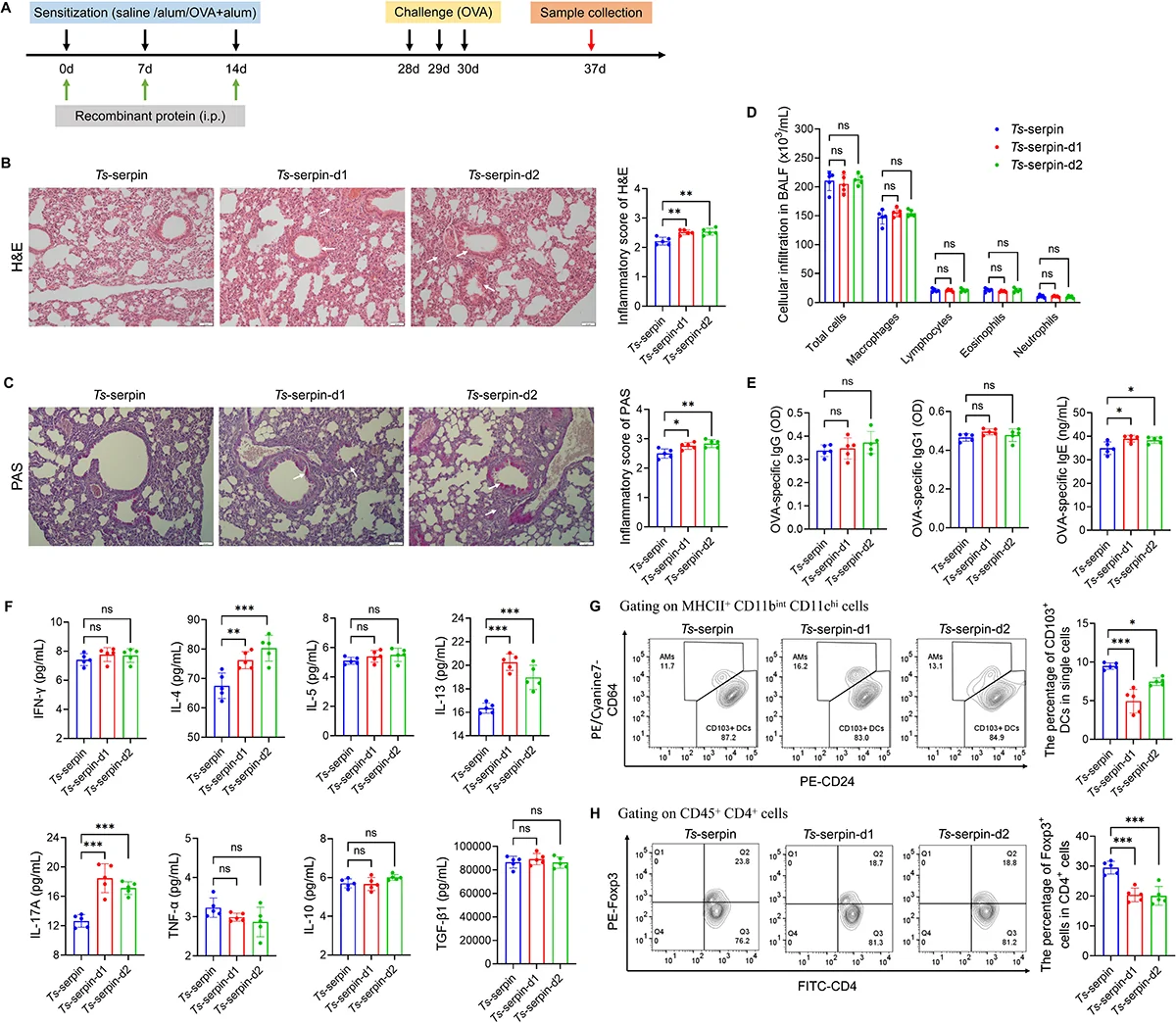
The enzyme inhibitory activity of *Ts*-serpin slightly suppresses allergic inflammation in the lung at the sensitization stage.

To elucidate the mechanism by which *Ts*-serpin exerts an inhibitory effect during the sensitization stage, we distinguished immunoregulation mediated by enzyme inhibitory activity via intervention with mutant proteins (Figure 3(A)). Amino acid point mutation weakened the inhibitory effect of *Ts*-serpin on chymotrypsin and elastase, and the two mutant proteins showed a preference for protease inhibition (Figure S3). Mild inflammation and an increased number of goblet cells were observed in the lung tissue of the mice treated with the mutant proteins (Figure 3(B,C)). No significant change in the number of cells or the proportion of cell subsets in the BALF were noted among the groups treated with different proteins (Figure 3(D)). Antibody test results revealed that serum OVA-specific IgE levels increased to a less extent in the mutant protein intervention groups, whereas the levels of IgG and IgG1 did not change (Figure 3(E)). Notably, the serum levels of the cytokines IL-4, IL-13 and IL-17A increased in the mutant protein intervention groups (Figure 3(F)). Mice treated with the mutant proteins presented a reduced proportion of CD103^+^ DCs in lung tissue (Figure 3(G)) and a reduced proportion of Treg cells in the spleen (Figure 3(H)), but no significant effect on the proportions of other immune cells (Figure S4(A-J)). Taken together, these results indicate that despite partially suppressing the systemic type 2 immune response, the intervention of the enzyme inhibitory activity of *Ts*-serpin at the sensitization stage, was unable to play a leading role in preventing the occurrence of allergic inflammation.

(A) The intervention time points of *Ts*-serpin/*Ts*-serpin-d1/*Ts*-serpin-d2 at the sensitization stage. (B) H&E staining results of the mouse lung in the recombinant protein intervention groups. Magnification: 200 x . (C) PAS staining results of the mouse lung in the recombinant protein intervention groups. Magnification: 200 x . (D) Statistical plot of the number of various immune cell subsets in the BALF. (E) The OVA-specific antibody levels (IgG, IgG1, IgE) in the serum. (F) The cytokine levels in the serum. (G) Plots and cell proportion statistics of CD103^+^ DCs in the mouse lung. (H) Plots and cell proportion statistics of Treg cells in the mouse spleen. The sample size for all data was five.

### The enzyme inhibitory activity of *Ts*-serpin dominates the suppressive effect during intervention at the challenge stage of the acute allergy model

**Figure 4.**
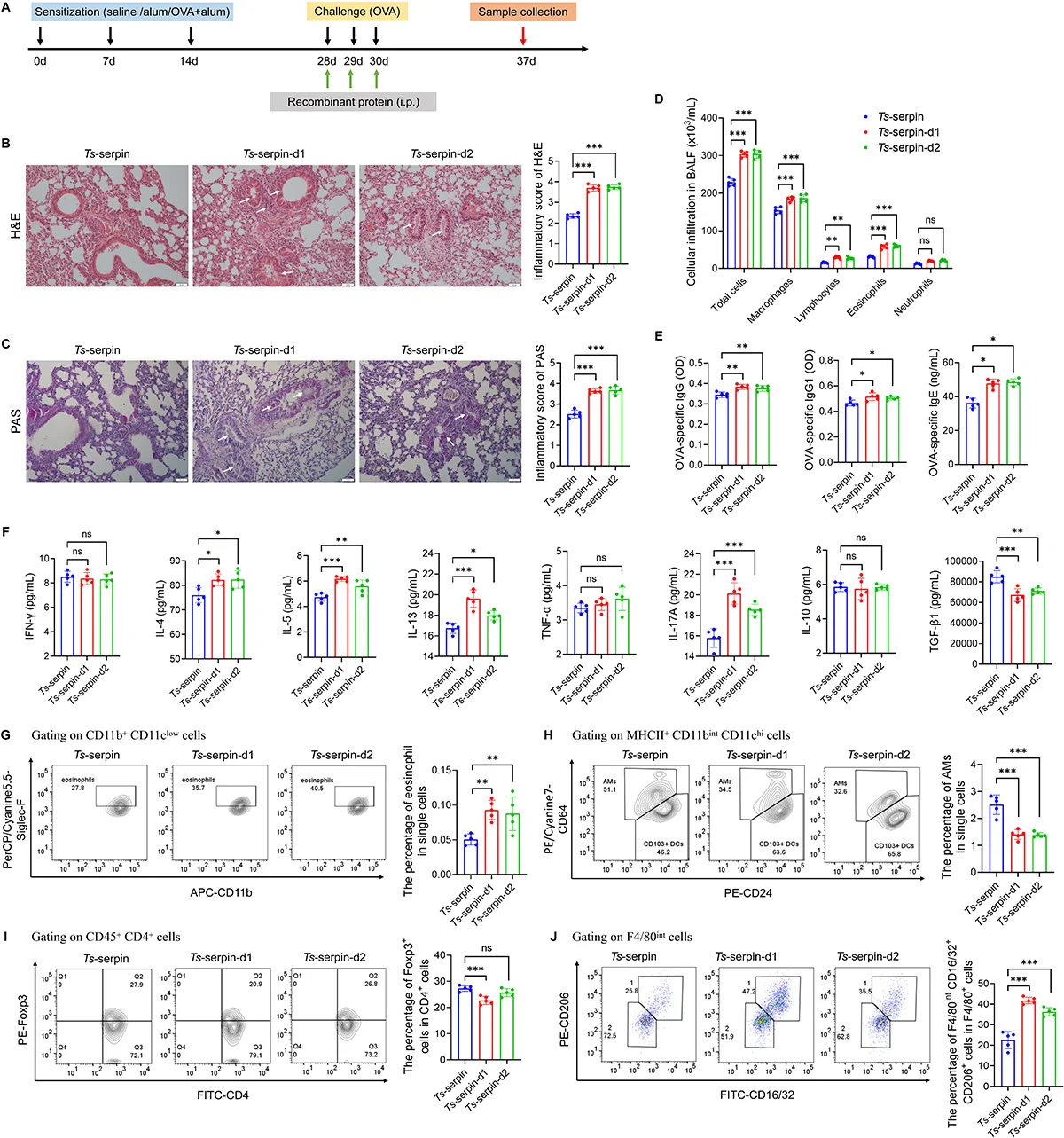
The enzyme inhibitory activity of *Ts*-serpin significantly ameliorates allergic inflammation in the lung at the challenge stage.

To further elucidate the molecular mechanism of immune tolerance induced by *Ts*-serpin at the challenge stage, we adopted the method of mutant protein intervention (Figure 4(A)). Compared with those of the *Ts*-serpin-treated mice, the lung tissues of the mice treated with the mutant proteins presented significantly more severe damage and increased bronchial mucus and numbers of goblet cells (Figure 4(B,C)). Consistent with these findings, in the context of mutant protein intervention, the BALF presented significantly increased total cell, macrophage, lymphocyte, and eosinophil counts (Figure 4(D)), and the levels of OVA-specific antibodies (IgG, IgG1, and IgE) were greater than those in *Ts*-serpin treated mice (Figure 4(E)). Compared with those in the *Ts*-serpin intervention group, the cytokine changes in the group of mice treated with mutant proteins revealed that the levels of type 2 immune-and Th17 immune-related cytokines were significantly greater, whereas the TGF-β1 level was significantly lower (Figure 4(F)). Additionally, the proportion of eosinophils in the lung tissue of mice treated with mutant proteins significantly increased (Figure 4(G)), whereas the proportion of AMs decreased significantly (Figure 4(H)). Interestingly, the proportions of Treg cells in the *Ts*-serpin-d1 and *Ts*-serpin-d2 intervention groups were significantly different, and the proportion of Treg cells in the *Ts*-serpin-d1 intervention group was significantly lower (Figure 4(I)). Mutated protein intervention significantly induced high expression of CD206 and CD16/32 in F4/80^int^ peritoneal macrophages (Figure 4(J)). Compared with *Ts*-serpin, mutated protein intervention did not affect the proportions of IMs, CD11b^+^ DCs, CD103^+^ DCs, CD45^+^ cells, B220^+^CD4^−^ B cells, F4/80^+^ total macrophages, F4/80^hi^ macrophages, or F4/80^int^ macrophages (Figure S5(A-H)). Together, these data suggest that rapid regulatory pathways mediated by enzymatic responses are key to establishing immune tolerance in multiple tissues during intervention in the challenge stage of allergic disease.

(A) The intervention time points of *Ts*-serpin/*Ts*-serpin-d1/*Ts*-serpin-d2 at the challenge stage. (B) H&E staining results of the mouse lung in the recombinant protein intervention groups. Magnification: 200 x . (C) PAS staining results of the mouse lung in the recombinant protein intervention groups. Magnification: 200 x . (D) Statistical plot of the number of various immune cell subsets in the BALF. (E) The OVA-specific antibody levels (IgG, IgG1, IgE) in the serum. (F) The cytokine levels in the serum. (G) Plots and cell proportion statistics of eosinophils in the mouse lung. (H) Plots and cell proportion statistics of AMs in the mouse lung. (I) Plots and cell proportion statistics of Treg cells in the mouse spleen. (J) Plots and cell proportion statistics of F4/80^int^ CD16/32^+^ CD206^+^ macrophages in the mouse peritoneal cavity. The sample size for all data was five.

### The enzyme inhibitory activity of *Ts*-serpin partially suppresses inflammation during intervention at the treatment stage of the acute allergy model

**Figure 5.**
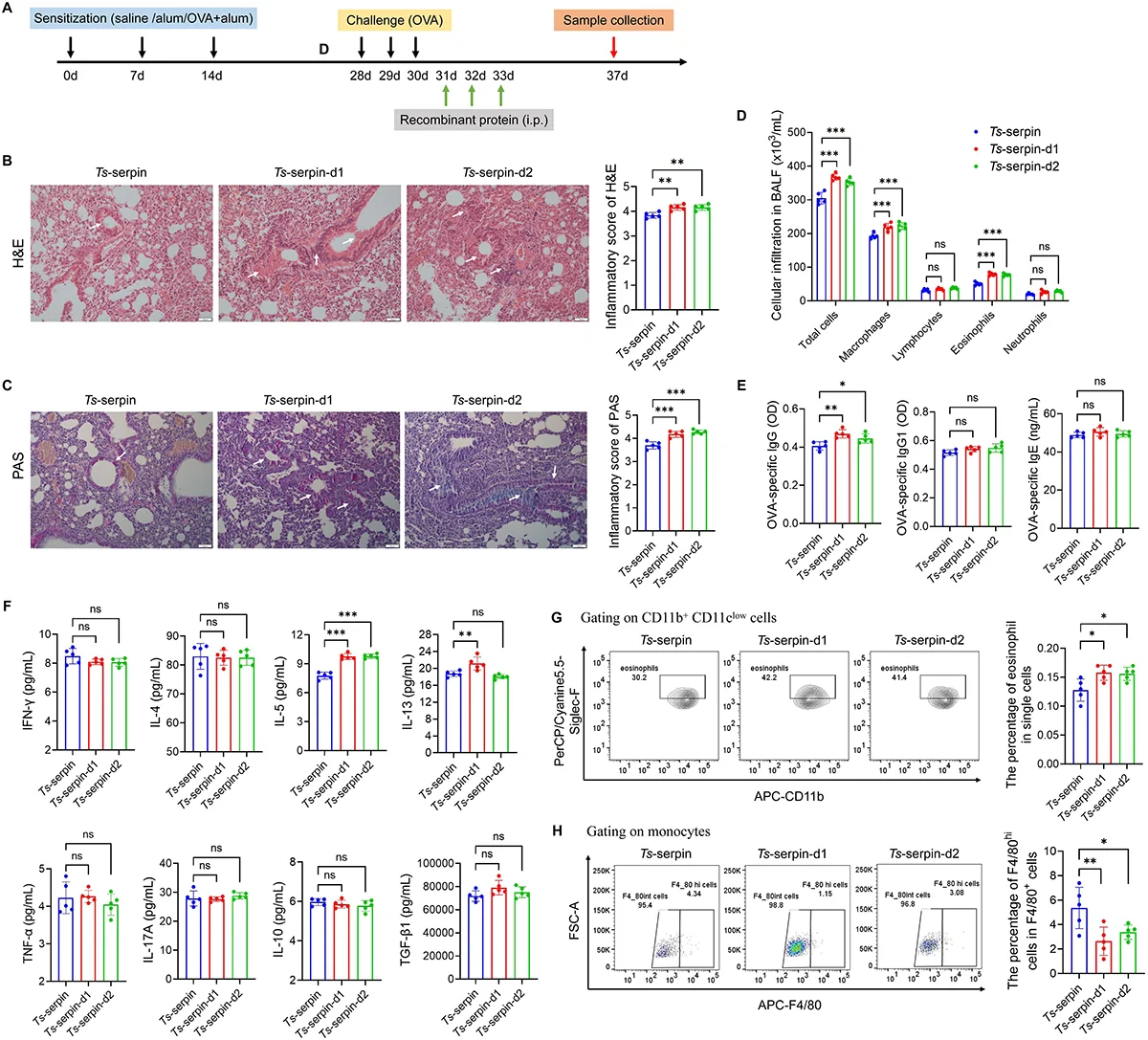
The enzyme inhibitory activity of *Ts*-serpin partially suppresses allergic inflammation in the lung at the treatment stage.

Mutated proteins were used to investigate the therapeutic effect of enzyme inhibitory activity at the treatment stage of allergic diseases (Figure 5(A)). Compared with the *Ts*-serpin intervention group, the mutant protein intervention groups presented more severe lung pathological injury (Figure 5(B,C)). Consistently, the number of total cells, macrophages and eosinophils in the BALF increased significantly in the mutant protein intervention groups (Figure 5(D)). The decrease in enzyme inhibitory activity only increased specific IgG levels in the mutant protein intervention groups, without changing IgG1 and IgE levels (Figure 5(E)). Compared with those in the *Ts*-serpin intervention group, the IL-5 levels in the mutant protein intervention groups were increased. However, only the IL-13 level in the *Ts*-serpin-d1 intervention group was increased, and the levels of other cytokines did not significantly change (Figure 5(F)). Interestingly, the *Ts*-serpin-d1 and *Ts*-serpin-d2 intervention groups presented increases in eosinophils in the lung tissue and a lower proportion of F4/80^hi^ macrophages in the peritoneal cavity than the *Ts*-serpin intervention group did (Figure 5(G,H)). The enzyme inhibitory activity affected the proportions of F4/80^+^ macrophages and F4/80^int^ macrophages in the peritoneal cavity, whereas the other cells were not affected (Figure S6(A-J)). Together, these data suggest that the enzyme inhibitory activity of *Ts*-serpin partially ameliorates pathological injury to lung tissue by inducing peritoneal macrophage differentiation during intervention in the treatment of allergic disease.

(A) The intervention time points of *Ts*-serpin/*Ts*-serpin-d1/*Ts*-serpin-d2 at the treatment stage. (B) H&E staining results of the mouse lung in the recombinant protein intervention groups. Magnification: 200 x . (C) PAS staining results of the mouse lung in the recombinant protein intervention groups. Magnification: 200 x . (D) Statistical plot of the number of various immune cell subsets in the BALF. (E) The OVA-specific antibody levels (IgG, IgG1, IgE) in the serum. (F) The cytokine levels in the serum. (G) Plots and cell proportion statistics of eosinophils in the mouse lung. (H) Plots and cell proportion statistics of F4/80^hi^ macrophages in the mouse peritoneal cavity. The sample size for all data was five.

The 33d of the timeline in Figure 5 (A) was repeatedly written as 32d, and we uploaded the revised picture without other changes.

### The enzyme inhibitory activity of *Ts*-serpin is important to induce Treg cell differentiation

**Figure 6.**
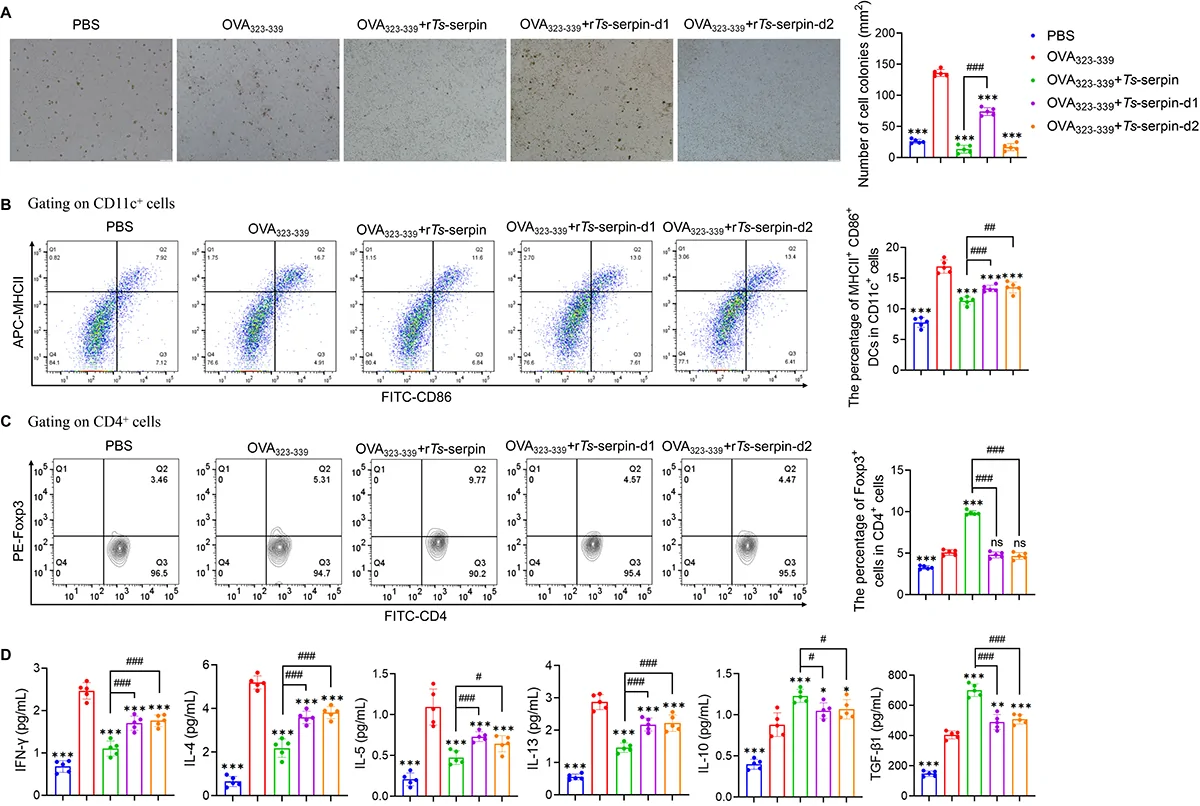
The enzyme inhibitory activity of *Ts*-serpin is important to induce the Treg cell differentiation *in vitro*.

Coculture with primary cells *in vitro* was used to examine the effect of enzyme inhibitory activity on the differentiation of naïve CD4^+^ T cells. Compared with those in the PBS control group, OVA_323–339_-treated BMDCs formed clearly visible colonies with CD4^+^ T cells. Interestingly, colony formation was significantly inhibited in the group treated with *Ts*-serpin and was partially restored by *Ts*-serpin-d1 but not *Ts*-serpin-d2 (Figure 6(A)). The proportion of MHCII^+^ CD86^+^ BMDCs were significantly inhibited by *Ts*-serpin, and the inhibitory ability of the *Ts*-serpin-d1/*Ts*-serpin-d2 was weakened (Figure 6(B)). Additionally, the mutant proteins did not induce a high proportion of Treg cells in contrast to that noted for *Ts*-serpin (Figure 6(C)). The levels of anti-inflammatory cytokines IL-10 and TGF-β1 in the supernatant of cell culture were significantly increased after *Ts*-serpin intervention, and the levels of other inflammatory cytokines were inhibited. This inhibition was weakened with the decrease of enzyme inhibitory activity (Figure 6(D)). The differences in cell colony formation and T-cell differentiation between the mutant protein-treated groups suggest that a bias in enzyme inhibitory activity may mediates different induction mechanisms. The enzyme inhibitory activity of *Ts*-serpin is important for regulating BMDCs to promote Treg cells differentiation.

(A) Light microscopic images and quantitative statistics of colonies in the coculture system of BMDCs and naïve CD4^+^ T cells. Magnification: 100 x . (B, C) After 48 h of coculture, plots and cell proportion statistics of MHCII^+^ CD86^+^ DCs and CD4^+^ Foxp3^+^ Treg cells. (D) The levels of cytokines in the cell culture supernatant. The sample size for all data was five.

### The enzyme inhibitory activity of *Ts*-serpin affects macrophages to inhibit inflammation

**Figure 7.**
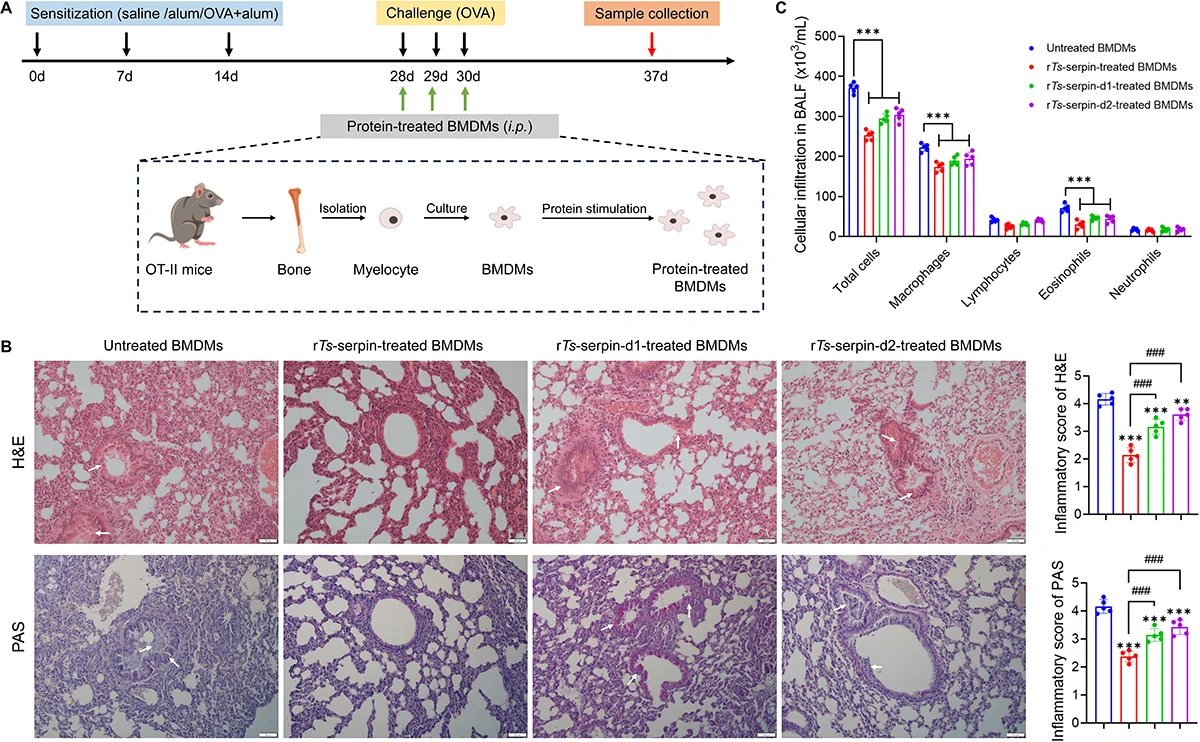
The enzyme inhibitory activity of *Ts*-serpin regulates BMDMs to inhibit allergic inflammation.

The *in vivo* experiment showed that the most significant intervention effect could be obtained when the proteins were treated at the allergen challenge stage, and also verified its potential value in clinical translation. Meanwhile, the results of the mutant protein intervention groups suggested that macrophages were the key immune cell population that was most significantly regulated by the enzyme inhibitory activity. Based on this, we further constructed an *in vitro* macrophage culture system and an adoptive transfer model to directly verify the anti-inflammatory effect of protein-treated macrophages (Figure 7(A)). The results of lung showed that BMDMs incubated with protein exhibited obvious anti-inflammatory properties. Compared with r*Ts*-serpin-treated BMDMs intervention group, mutant protein-treated BMDMs intervention groups showed more obvious pathological changes in lung tissue, such as inflammatory cell infiltration, thickening of bronchial walls, inflammatory exudates in bronchi, and goblet cell proliferation (Figure 7(B)). Meanwhile, the number of cells in BALF of mice intervened with the mutant protein-treated BMDMs was also significantly increased (Figure 7(C)).

(A) Schematic diagram of BMDMs isolation, culture, and adoptive transfer. (B) H&E and PAS staining results of mouse lung in the BMDMs intervention groups. Magnification: 200 x . (C) Statistical plot of the number of various immune cell subsets in the BALF. The sample size for all data was five.

## Discussion

The sensitization and challenge of allergens are indispensable processes for inducing hypersensitivity. The development of the sensitization stage in clinical patients usually occurs in childhood, and the onset time is uncontrollable [20,21]. Therefore, current clinical treatments usually focus on removing the allergen or blocking the key inflammatory molecules produced in the challenge stage, such as histamine, IgE, and IL-5 [22,23]. However, neither physical isolation nor biological therapy can correct the allergen-mediated immune imbalance, and both are associated with nonnegligible side effects, such as central nervous system depression, anticholinergic effects, anaphylaxis, and injection-site reactions [24–26]. Worms are successful manipulators of the host immune system, given the variety of worm-derived immunomodulators [27,28]. Serpins are a class of immunomodulators that are commonly secreted by worms [29,30], and have good therapeutic potential for immune imbalance diseases; however, previous studies often confused the immunoregulation mediated by protein structure and enzyme inhibitory activity. Elucidating the immunoregulatory mechanism of a single immunomodulator is the basis for the safe and accurate application of helminth therapy [31,32]. Worm-derived proteins interact with pathogen-associated molecular patterns on the surface of immune cells to activate the innate immune system, yielding a range of immunoregulatory effects [33]. Differently, enzyme inhibitory activity directly interferes with the host enzymatic reaction, thereby regulating the immune system, and this response is characterized by high efficiency, rapidity and extensiveness [34,35]. In this study, we evaluated the importance of the enzyme inhibitory activities of *T. spiralis*-derived serine protease inhibitor in the induction of immune tolerance in an OVA-induced acute allergy model. Specifically, we reduced the enzyme inhibitory activity of *Ts*-serpin by protein mutation, and investigated the immunomodulatory effects at different stages of allergic disease and the relevant cellular regulatory network.

The major immune cell populations involved in the immunoregulatory effects mediated by the enzyme inhibitory activity of the *Ts*-serpin vary greatly at different stages of the allergic diseases. The effect of enzyme inhibition on peritoneal macrophages was time- sensitive, and the regulatory effect weakened with increasing protein stimulation time, especially for F4/80^hi^ macrophages. Elastase not only impairs the phagocytic function of macrophages by cleaving membrane surface proteins, including low-density lipoprotein receptor-associated protein 1 (LRP1) and calcium-regulated calcium ion-dependent papain-like cysteine protease-2 (calpain-2) but also selectively targets membrane-bound proteoglycans to affect the migration and polarization of macrophages [36,37]. In particular, the neuropilin-1 protein cleaved by elastase is crucial for microglial phagocytosis and the modulation of the immunosuppressive response, and it is also required for tumor-associated macrophage migration [38]. Similarly, the number of peritoneal macrophages was significantly reduced during the treatment stage of *Ts*-serpin-d2 intervention in our study, suggesting that increased elastase activity impaired the proportion of macrophages. In addition, we found that peritoneal macrophages were preferentially involved in the establishment of systemic immune tolerance over splenic Treg cells in terms of engagement timing. In contrast, the regulatory effect of *Ts*-serpin on Treg cells gradually became evident over time, and the enzyme inhibitory activity of *Ts*-serpin was important to induce Treg cells differentiation. Chymotrypsin is a potent chemotactic molecule for lymphocytes and monocytes [39], and this feature is consistent with our results of the formation of cell colonies by BMDCs and T cells *in vitro* cell coculture experiments. In addition, blocking chymotrypsin activity impaired DC phagocytosis and the ability to activate T cells [40], which may affect the effectiveness of OVA sensitization. This may explain why the attenuated enzyme inhibitory activity during the intervention in the sensitization process slightly enhanced the type 2 immune response and lung tissue injury. Interestingly, it appears that immune tolerance established by protein during the sensitization stage is not primarily mediated by Treg cells induced through enzyme inhibitory activity, but rather may have affected the antigen presentation process to reduce the memory of the immune system to OVA. Additionally, neutrophil-derived elastase induces CD4^+^ T cells to differentiate into proinflammatory phenotypes [41], representing a possible factor for the increase in type 2 immune-related cytokines in the mutant protein intervention groups. Although our data show that *Ts*-serpin induces a potent immune tolerance response, the molecular mechanism has not been fully elucidated. At present, it is uncertain whether this effect involves stable epigenetic reprogramming (e.g. TSDR demethylation in Treg cells [42], histone modifications, or chromatin remodeling in macrophages and DC [43]) or reflects only a transient anti-inflammatory response. In the future, multi-omics approaches (single-cell transcriptome, ATAC-seq and methylation microarray) are needed to clarify whether *Ts*-serpin truly establishes a long-term, genetically encoded tolerance state. These results indicate that the enzymatic inhibitory activity of *Ts*-serpin regulates immune cells, and highlight the importance of enzyme inhibitory activity for the rapid restoration of immune imbalance.

Systemic immune tolerance may change the immune microenvironment to maintain immune homeostasis in the lung by regulating innate immune cells. AMs and CD103^+^ DCs, two representative immune cells that inhibit excessive inflammation in the lung [44,45], were increased to varying degrees during the sensitization and challenge stages of *Ts*-serpin intervention and were significantly suppressed in mutant protein intervention groups, suggesting a correlation with enzyme inhibitory activity. Interestingly, the change trends of AMs and CD103^+^ DCs are consistent with our previous study with *T. spiralis* adult intervention [8]. Although the precise regulatory mechanisms remain unknown, this study confirmed that the enzyme inhibitory activity of *Ts*-serpin contributes to this process. The effectiveness of enzyme inhibition in maintaining immune cell homeostasis in the lung is limited to the period before lung tissue inflammation is fully activated. Therefore, the future therapeutic clinical application of *Ts*-serpin-established immune tolerance is more likely to be used in the early stage of allergen challenge.

In addition to classical Th2-type cytokines, this study also showed that *Ts*-serpin significantly reduced IL-17A levels in the serum, whereas the mutant protein intervention groups exhibited elevated IL-17A levels, a finding with important pathological and translational implications. As a key driver of barrier immunity and autoimmunity, IL-17A in asthma attracts neutrophils to the airway by promoting the production of neutrophil chemokines, such as CXCL1 and CXCL8, which drives the neutrophilic asthma phenotype with more severe inflammation, glucocorticoid insensitivity and worse prognosis [46,47]. The downregulation of IL-17A by *Ts*-serpin suggests that *Ts*-serpin may have therapeutic potential not only in Th2-driven allergic asthma, but also in severe asthma, mixed asthma and autoimmune airway diseases. The effect of *Ts*-serpin on IL-17A expression suggests that the immunoregulatory effect of *Ts*-serpin is partly dependent on its enzyme inhibitory activity, which may affect the signaling pathway related to Th17 differentiation [48], or alter the secretion of IL-17A by innate immune cells [49]. However, the current data cannot distinguish whether *Ts*-serpin directly affects the differentiation of Th17 cells, or indirectly affects the production of IL-17A. In the future, it needs to be systematically analyzed by means of *in vitro* Th17 differentiation system, proteomics and single-cell transcriptomics.

TGF-β1 has broad-spectrum immunosuppressive properties and can negatively regulate the immune response of Th1, Th2 and Th17 at the same time [50], which is consistent with the comprehensive and significant down-regulation trend of Th1/Th2/Th17 cytokines observed in the *Ts*-serpin intervention group in this study. Mechanistically, TGF-β1 induces the differentiation of Foxp3^+^ Treg cells and directly inhibits the proliferation and differentiation of effector T cells through the Smad signaling pathway [51]. Unlike IL-10, which acts primarily as a feedback suppressor of “ending inflammation,” TGF-β1 is more likely to act as a homeostatic maintenance factor that continuously constrains the activation threshold of T cells at lower levels [52]. It is worth noting that broad-spectrum immunosuppression without IL-10 elevation is not a rare phenomenon, and TGF-β1-dominated immune microenvironment or T cell anergy can also mediate such suppression [53]. *Ts*-serpin, as a serine protease inhibitor, may interfere with the protease activity on the surface of antigen-presenting cells, thereby changing the stability of MHC-peptide complexes or weakening the TCR signal strength, and finally inducing T cell anergy [54]. Although this study confirmed the functional association between the enzyme inhibitory activity of *Ts*-serpin and phenotypic changes of DCs, the regulation of MHC-II peptide repertoire formation and tolerogenic signaling pathways was not examined. Taken together, the broad-spectrum immunosuppression induced by *Ts*-serpin may involve multiple pathways in concert rather than relying on a single cytokine or signaling axis.

However, this study has several serious limitations. (I) The OVA-induced OT-II mouse acute allergy model is mediated by an acute Th2-skewed response, which cannot fully simulate the genetic, environmental, microbial and chronic multi-factor complexity of human allergic diseases. Therefore, the clinical translational significance of *Ts*-serpin immune tolerance remains uncertain. (II) The long-term durability and safety of *Ts*-serpin-induced tolerance have not been evaluated, and the establishment of tolerance may impair the ability of the host to resist pathogen infection, which has potential clinical drawbacks. (III) Due to the lack of multi-omics methods such as RNA-seq, proteomics, metabolomics and single-cell analysis, the molecular pathways, regulatory networks and cell-specific signaling events of *Ts*-serpin-mediated immune tolerance have not been fully analyzed. (IV) The immunogenicity and protein targeting of *Ts*-serpin in the clinical setting have not been validated. (V) Although the intraperitoneal injection method in this study is conducive to the mechanism verification, the future clinical transformation needs to further evaluate the closer to clinical delivery route and its influence on the efficacy, such as aerosol inhalation or subcutaneous injection. (VI) Disease model selected OVA is a non-environmental, non-inhalant allergen. There is a lack of validation of clinically relevant environmental allergen, such as house dust mites.

By introducing amino acid point mutations to weaken the enzyme inhibitory activity of *Ts*-serpin, we investigated the therapeutic effect on enzyme inhibitory activity at different developmental stages of OVA-induced acute allergy model. The establishment of immune tolerance by protein-regulated macrophages or Treg cells is the key to rapid therapeutic intervention. Our study not only provides new ideas for elucidating the immunomodulatory mechanisms of serine protease inhibitor activity, but also provides new insights into the treatment of allergic diseases by enzymatic reactions.

## Supplementary Material

Supporting Information.docx

## Data Availability

The raw data and supplementary material are acquired from Science Data Bank (DOI: https://doi.org/10.57760/sciencedb.37394) [55].

## Abbreviations

Serpins: serine protease inhibitors; OVA: ovalbumin; IMs: interstitial macrophages; AMs: alveolar macrophages; Treg cells: regulatory T cells; BALF: bronchoalveolar lavage fluid; LRP1: lipoprotein receptor-associated protein 1; calpain-2: papain-like cysteine protease-2; H&E: hematoxylin and eosin.

## ARRIVE guidelines

The manuscript has adhered to ARRIVE guidelines.
