# Supplementary material for: The enzyme inhibitory activity of helminth-derived serine protease inhibitor rapidly establishes immune tolerance to ameliorate allergic diseases
Source: Virulence. 2026 Sep 6;17(1):2725991. doi: 10.1080/21505594.2026.2725991 (PMC13556981; doi:10.1080/21505594.2026.2725991)
Supplement: Supplementary file 1 [file KVIR_A_2725991_SM2697.docx]

**Supplementary material:**

**Supplemental Figure**

**Figure S1. Coomassie brilliant blue staining images of recombinant proteins.**

**Figure S2. The proportion of immune cell subsets at the three stages of OVA-induced asthma model intervened with *Ts*-serpin.**

**Figure S3. The inhibitory effects of the recombinant protein on chymotrypsin and elastase.**

(A) Absorbance at 410 nm of chromogenic substrate in solution after incubation of recombinant proteins with chymotrypsin. (B) Absorbance at 405 nm of chromogenic substrate in solution after incubation of recombinant proteins with elastase.

**Figure S4. Proportion statistics of immune cell subsets during the sensitization stage of OVA-induced asthma model intervened with *Ts*-serpin and mutant proteins.**

**Figure S5. Proportion statistics of immune cell subsets during the challenge stage of OVA-induced asthma model intervened with *Ts*-serpin and mutant proteins.**

**Figure S6. Proportion statistics of immune cell subsets during the treatment stage of OVA-induced asthma model intervened with *Ts*-serpin and mutant proteins.**

**Supplemental Table**

**Table S1. Experimental reagent and equipment**

| Reagent/ Equipment | Cat |
| --- | --- |
| OVA_323-339_ | O1641 (Sigma-Aldrich, St. Louis, Missouri, USA) |
| OVA | S7951 (Sigma-Aldrich, St. Louis, Missouri, USA) |
| Chymotrypsin | C2160000 (Sigma-Aldrich, St. Louis, Missouri, USA) |
| Elastase (porcine pancreas) | E0258 (Sigma-Aldrich, St. Louis, Missouri, USA) |
| Protease substrate (chymotrypsin) | S2628 (Sigma-Aldrich, St. Louis, Missouri, USA) |
| Protease substrate (elastase) | S4760 (Sigma-Aldrich, St. Louis, Missouri, USA) |
| Imject alum adjuvant | 77161 (Thermo Fisher Scientific, Waltham, Massachusetts) |
| Diff-Quick Stain Kit | G1540 (Solarbio life sciences, Beijing, China) |
| Mouse TGF-β1 ELISA Kit | abs552208 (Absin, Beijing, China) |
| Mouse ovalbumin IgE Antibody ELISA kit | MM-45931M2 (Mei mian, Jiangsu, China) |
| Red Blood Cell Lysis | R1010 (Solarbio life sciences, Beijing, China) |
| Recombinant mouse GM-CSF | 315-03 (PeproTech, Cranbury, NJ, USA) |
| Recombinant mouse IL-4 | 214-14 (PeproTech, Cranbury, NJ, USA) |
| Recombinant mouse M-CSF | 315-02 (PeproTech, Cranbury, NJ, USA) |
| EasySep^TM^ mouse Naïve CD4^+^ T-cell isolation kits | 19765 (STEMCELL, Vancouver, BC, Canada) |
| Mouse BD Fc Block antibody | 553141 (BD Biosciences, Franklin Lakes, NJ, USA) |
| PE Rat anti-mouse Foxp3 | 563101 (BD Biosciences, Franklin Lakes, NJ, USA) |
| APC Rat anti-mouse CD19 | 115512 (BioLegend, San Diego, CA) |
| APC Rat anti-mouse F4/80 | 123116 (BioLegend, San Diego, CA) |
| FITC Rat anti-mouse CD16/32 | 156614 (BioLegend, San Diego, CA) |
| PE Goat anti-mouse CD206 | FAB2535P (R&D Systems, Minnesota) |
| PerCP/Cyanine5.5 Rat anti-mouse CD45 | 103132 (BioLegend, San Diego, CA) |
| APC Rat anti-mouse CD45R/B220 | 103212 (BioLegend, San Diego, CA) |
| FITC Rat anti-mouse CD4 | 100406 (BioLegend, San Diego, CA) |
| PE/Cyanine7 Rat anti-mouse CD64 | 164414 (BioLegend, San Diego, CA) |
| PerCP/Cyanine5.5 anti-mouse I-A/I-E | 107625 (BioLegend, San Diego, CA) |
| PerCP/Cyanine5.5 anti-mouse CD170 (Siglec-F) | 155525 (BioLegend, San Diego, CA) |
| Reagent/ Equipment | Cat |
| APC anti-mouse/human CD11b | 101212 (BioLegend, San Diego, CA) |
| FITC anti-mouse CD11c | 117305 (BioLegend, San Diego, CA) |
| PE anti-mouse CD11c | 117307 (BioLegend, San Diego, CA) |
| FITC anti-mouse CD86 | 105005 (BioLegend, San Diego, CA) |
| APC anti-mouse MHCII | 107614 (BioLegend, San Diego, CA) |
| PE anti-mouse Ly-6G | 127607 (BioLegend, San Diego, CA) |
| PE anti-mouse CD24 | 101807 (BioLegend, San Diego, CA) |
| Goat anti-mouse IgG (HRP) | ab6789 (Abcam, Cambridge, United Kingdom) |
| Goat anti-mouse IgG1 (HRP) | ab97240 (Abcam Cambridge, United Kingdom) |
| BD FACSVerse^TM^ flow cytometry | BD Biosciences, Franklin Lakes, NJ, USA |
| Biotek epoch microplate reader | BioTek Instruments, Winooski, Vermont, USA |
| AKTA protein purification system | Cytiva, Marlborough, Massachusetts, USA |
